# Surveillance of Tahyna Orthobunyavirus in Urban Areas in Croatia—The “One Health” Approach

**DOI:** 10.3390/tropicalmed7100320

**Published:** 2022-10-20

**Authors:** Vladimir Stevanovic, Tatjana Vilibic-Cavlek, Vladimir Savic, Ana Klobucar, Snjezana Kovac, Marcela Curman Posavec, Suncica Petrinic, Maja Bogdanic, Marija Santini, Vanja Tesic, Nathalia de Albuquerque Soares, Ljubo Barbic

**Affiliations:** 1 Department of Microbiology and Infectious Diseases with Clinic, Faculty of Veterinary Medicine, University of Zagreb, 10000 Zagreb, Croatia; 2 Department of Virology, Croatian Institute of Public Health, 10000 Zagreb, Croatia; 3 Department of Microbiology, School of Medicine, University of Zagreb, 10000 Zagreb, Croatia; 4 Poultry Center, Croatian Veterinary Institute, 10000 Zagreb, Croatia; 5 Department of Epidemiology, Andrija Stampar Teaching Institute of Public Health, 10000 Zagreb, Croatia; 6 Department for Adult Intensive Care and Neuroinfections, University Hospital for Infectious Diseases “Dr. Fran Mihaljevic”, 10000 Zagreb, Croatia; 7 Department of Social Medicine and Epidemiology, Medical Faculty, University of Rijeka, 51000 Rijeka, Croatia

**Keywords:** Tahyna orthobunyavirus, humans, horses, pet animals, mosquitoes, Croatia, One Health

## Abstract

Background: Tahyna orthobunyavirus (TAHV) is a neglected mosquito-borne bunyavirus. Although the virus is widespread in continental Europe, TAHV infections are rarely reported. We analyzed the prevalence of TAHV in humans and different animal species as well as mosquitoes collected in urban areas of Zagreb and its surroundings in the period from 2020 to 2022. Methods: The study included 32 patients with neuroinvasive disease (NID), 218 asymptomatic individuals, 98 horses, 94 pet animals (dogs and cats), and 4456 *Aedes vexans* mosquitoes. Cerebrospinal fluid (CSF) and urine samples of patients with NID were tested for the TAHV RNA using a real-time reverse transcription-polymerase chain reaction (RT-qPCR). Human and animal serum samples were tested for TAHV-neutralizing (NT) antibodies using a virus-neutralization test (VNT). Mosquito pools were tested for TAHV RNA using an RT-qPCR. Results: TAHV NT antibodies were detected in 3/9.4% of patients with NID, 8/3.7% of asymptomatic individuals, 29/29.6% of horses, and 11/11.7% of pet animals. There was no difference in the seroprevalence according to age, sex, and area of residence in asymptomatic individuals. In addition, TAHV seropositivity did not differ according to age and sex in pet animals. None of the tested mosquito pools was TAHV RNA-positive. Conclusions: The presented results highlight the importance of interdisciplinary surveillance (“One Health”) of this neglected viral zoonosis.

## 1. Introduction

Tahyna orthobunyavirus (TAHV) is an arthropod-borne virus of the family *Peribunyaviridae*, genus *Orthobunyavirus*, California encephalitis serogroup [1]. Hares, rabbits, hedgehogs, and rodents are vertebrate hosts for TAHV in a natural cycle, while floodwater mosquitoes *Aedes vexans* are the primary arthropod vectors. Humans represent only incidental or dead-end hosts for TAHV [2].

In humans, TAHV infections are typically asymptomatic. Symptomatic disease is usually presented as an influenza-like illness occurring in late summer and early autumn, mainly in children [3]. Fever, gastrointestinal disorders, atypical pneumonia, or myocarditis are the most common symptoms of TAHV infection [4]. Despite its association with neurovirulence (meningitis), TAHV infection is still an underdiagnosed disease, with only a few human clinical cases reported in recent decades [5].

TAHV is widespread throughout continental Europe, as evidenced by the virus detection and isolation from mosquitoes as well as the detection of antibodies in humans and animals [6,7,8]. High seroprevalence rates of up to 60–80% have been consistently observed among adult human populations of endemic foci, such as in the Czech Republic [9].

In addition, although transmission is focal and not uniform, seroprevalence studies conducted in wild ungulates in Austria, Hungary, and Romania have revealed that TAHV transmission to animals is widespread in Europe, particularly among wild boars, with a mean seroconversion rate of 15% [8]. In some high-endemic countries, such as the Czech Republic, TAHV antibodies were detected in various animal species with different seroprevalence rates: horses 34.4%, pigs 55%, cattle 5.6%, songbirds 14.0%, cormorants 22.6%, ducks 13.2%, mouflons 33.8%, deer 36.4–40.0%, wild boar 70.0%, and European hares 42.5% [9,10,11,12,13,14].

The presence of TAHV in mosquitoes (virus isolation or RNA detection) was confirmed in many countries including the Czech Republic [15], Slovakia [16], Italy [17], Austria [5,18], Russia [19], and China [20]. After the first isolation in Slovakia in 1958, TAHV was detected in several mosquito species, including *Ae. caspius*, *Ae. cinereus, Ae. dorsalis, Ae. cantans*, *Anopheles hyrcanus*, *Ae. punctor, Ae. communis, Ae. flavescens, Ae. excrucians*, *Oc. detritus* and *Culex pipiens, Cx. modestus*, and *Culiseta annulata* [5,7,20,21,22].

In Croatia, serologic evidence of TAHV dates back to the 1970s. Very rare seroprevalence studies showed TAHV antibodies in 7.9% of inhabitants of northeast Croatia and 0.2–1.47% of inhabitants of the Croatian littoral [23,24]. Only one study (1984–1988) detected TAHV hemagglutination-inhibiting antibodies in the serum samples of free-ranging European brown bears (*Ursus arctos*) collected within the Plitvice Lakes and Risnjak National Parks [25]. A more recently conducted Croatian study detected TAHV neutralizing (NT) antibodies in 10.1% of patients with unsolved neuroinvasive disease (NID) tested from 2017 to 2021, who developed symptoms during the arbovirus transmission season. In two patients presenting with meningitis, NT antibodies were also confirmed in the cerebrospinal fluid (CSF), suggesting a recent TAHV infection. The majority of seropositive patients (90.9%) were residents of floodplains along the rivers in continental Croatia; however, sporadic infections were also confirmed in the coastal region [26].

The aim of this study was to analyze the prevalence of TAHV in humans and different animal species as well as the virus detection in mosquitoes collected from urban areas of Zagreb and its surroundings.

## 2. Materials and Methods

### 2.1. Study Area

The study was conducted during a three-year period (May 2020–July 2022) and included patients with NID, asymptomatic individuals (seroprevalence investigation), pet animals (dogs, cats), horses, and mosquitoes. The sampling area included Zagreb, the capital of Croatia, and its surroundings. Zagreb is located in the northwest of the country on the banks of the river Sava (GPS coordinates 45°48′55.43″ N, 15°57′59.64″ E) at an elevation of approximately 112 m above sea level. The city is subdivided into 17 districts, of which the majority are located in the River Sava valley, at a low level. The city’s core region is densely built, while the northern part is situated on the Medvednica mountain’s slopes, with forest vegetation and smaller urban settlements. Agricultural lands dominate the eastern, southern, and western parts of the city area. The surface waterways abound in the city’s vicinity. Seven artificial lakes and several artificial watercourses are located in the city area.

Thirty-two mosquito species have been detected in the city of Zagreb. The species *Cx. pipiens* form molestus predominates in indoor breeding sites. Natural mosquito breeding sites in forested and flooded areas are active only in the spring (March–June), and the most common species are *Ae. sticticus*, *Ae. cantans*, *Ae. vexans*, and *Ae. geniculatus. Culex pipiens* is the most frequent mosquito species found in streams, while *Ae. albopictus* and *Cx. pipiens* are the most common mosquito species in artificial breeding sites [27,28].

**Figure 1:**
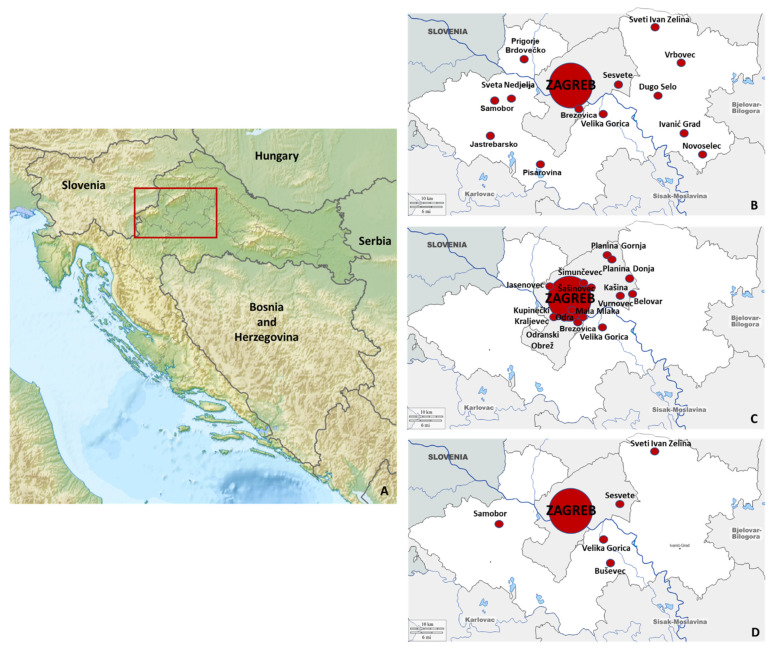
Sampling area in continental Croatia (**A**): human (**B**), horse (**C**), and pet animals (**D**) sampling areas.

The sampling area (human and animal sampling) in northwestern Croatia is presented in Figure 1.

### 2.2. Human Sampling and Testing

A total of 32 hospitalized patients presented with NID (febrile headache, meningitis, meningoencephalitis) were tested. Serum, CSF, and urine samples were collected from all patients. In addition, to determine the seroprevalence, a total of 218 serum samples from asymptomatic individuals were tested for the presence of TAHV NT antibodies. Samples were collected from patients during a routine check-up (part of physical examination, prior to surgery, prenatal testing, couples undergoing medically assisted reproduction). No participant reported a recent febrile disease.

CSF and urine samples of patients with NID were tested for the presence of TAHV RNA using a real-time reverse transcription polymerase chain reaction (RT-qPCR). The following primers and probes were used: forward primer: 5′-CCATTCCGTTAGGATCTTCTTCCT-3′, reverse primer 5′-CCTTCCTCTCCGGCTTACG-3′ and probe: FAM-5′-AATGCCGCAAAAGCCAAAGCTGC-3′-TAMRA [29].

TAHV antibodies were detected using a virus-neutralization test (VNT). UVE/TAHV/1958/CS/92 virus strain grown in Vero E6 cells was used as an antigen for the VNT. Virus titer (median tissue culture infectious dose; TCID_50_) was calculated using the Reed and Muench formula. Serum samples were heat-inactivated (30 min/56 °C) and diluted two-fold starting at 1:5. An equal amount of inactivated serum dilutions and 100 TCID_50_ of TAHV (25 µL) were mixed and incubated for one hour at 37 °C with CO_2_. Finally, 50 µL of 2 × 10^5^ Vero E6 cells/mL were added to each well. The plates were incubated at 37 °C with CO_2_ and inspected for the cytopathic effect after incubation for three days. NT antibody titer was defined as the reciprocal value of the highest serum dilution that showed at least 50% neutralization. Serum samples with neutralizing activity at dilutions ≥ 1:10 were considered seropositive [26].

### 2.3. Animal Sampling and Testing

Horses and pet animals (cats and dogs) were the animal species included in the study. Animal samples included 98 horse serum samples, 70 dog serum samples, and 24 cat serum samples from Zagreb and its surroundings. Sex and age were available for pet animals, while for horses, data were missing. TAHV VNT was performed as described above.

### 2.4. Mosquito Sampling and Testing

**Figure 2:**
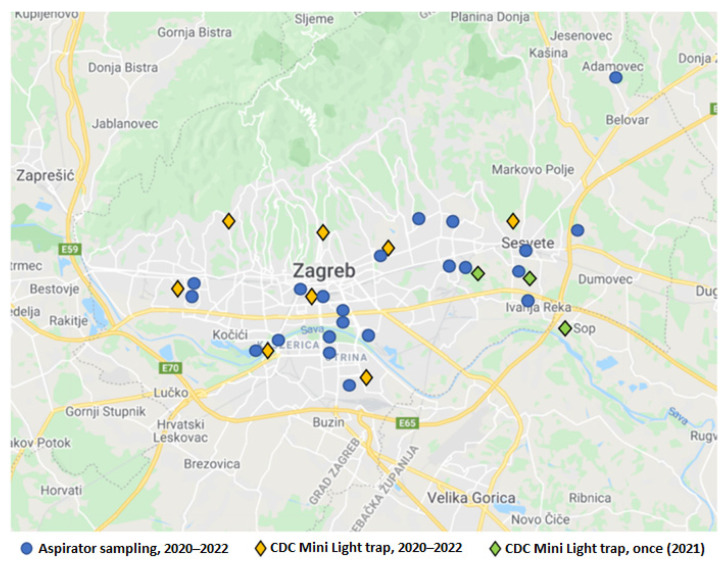
Distribution of mosquito sampling locations.

Mosquitoes were collected by two methods: CDC Mini Light traps (BioQuip, Products, Rancho Dominguez, CA, USA), and aspirator collection (human landing collection). CDC Mini Light traps were equipped with dry ice (CO_2_) as an attractant and used to collect adult mosquitoes. Traps were placed in the late afternoon before sunset, left overnight, and removed after sunrise (07:00–10:00). Over three years (2020–2022), the traps were set at the same eight collection sites (Figure 2, yellow marks) every 14 days, from May to October in 2020 and 2021 and from May to July in 2022. A total of 248 sampling occasions were gathered (2020: *n* = 96; 2021: *n* = 88; 2022: *n* = 64). Additionally, the sampling occasions using the CDC Mini Light trap were conducted once at three collection sites (Figure 2, green marks). During the same period, mosquito individuals were collected by aspirator as well as using the human landing collection method (Figure 2, blue dots). A total of 30 sampling occasions with *Ae. vexans* mosquitoes were gathered (2020: *n* = 18; 2021: *n* = 11; 2022: *n* = 1). Various habitats were selected for mosquito sampling: woods and gardens in the urban part of the city and populated areas close to the green belt.

The mosquitoes sampled by CDC Mini Light traps were transported to the laboratory in containers with dry ice, transferred to plastic tubes, and stored on dry ice until identification. Mosquitoes collected by the aspirator were transported alive to the laboratory in the aspirator, placed briefly in a freezer at −18 °C, and identified. Female mosquitoes were morphologically identified by species on a chilling surface under a stereomicroscope, using the determination keys by Becker et al. (2010) [30] and Schaffner et al. (2001) [31]. Specimens belonging to the same species collected on the same day and at the same sampling site were pooled, with up to 60 individuals per pool, and stored at −80 °C until virological testing. Only *Ae. vexans* individuals were tested.

Mosquito pools were tested for the presence of TAHV RNA as described above.

### 2.5. Statistical Analysis

The differences in seropositivity rates according to sex, age, and area of residence were compared using a chi-square or Fisher’s exact test. The strength of the association between dependent (VNT positivity) and independent variables was assessed by logistic regression. *p* < 0.05 was considered statistically significant. Statistical analysis was performed using Stata version 16 software.

## 3. Results

TAHV seroprevalence results are presented in Table 1. In humans, TAHV NT antibodies were detected in 3/32 (9.4%) patients with the neuroinvasive disease and 8/218 (3.7%) asymptomatic individuals. In addition, 29/98 (29.6%) of horses and 11/94 (11.7%) of pet animals were found to be TAHV-seropositive.

In asymptomatic persons, the seroprevalence rate was highest in 70+ year-olds (7.7%) compared to 2.5–3.8% in other age groups; however, this difference was not significant. No significant difference in seropositivity was found between males and females (3.7% vs. 3.6%) as well as among residents of urban and suburban areas (4.2% vs. 0%) (Table 2).

Results of the risk analysis in asymptomatic humans showed no association of TAHV seroprevalence with age, sex, and area of residence (Table 3).

In pet animals, there was no difference in the prevalence of TAHV NT antibodies according to age and sex (Table 4).

**Figure 3:**
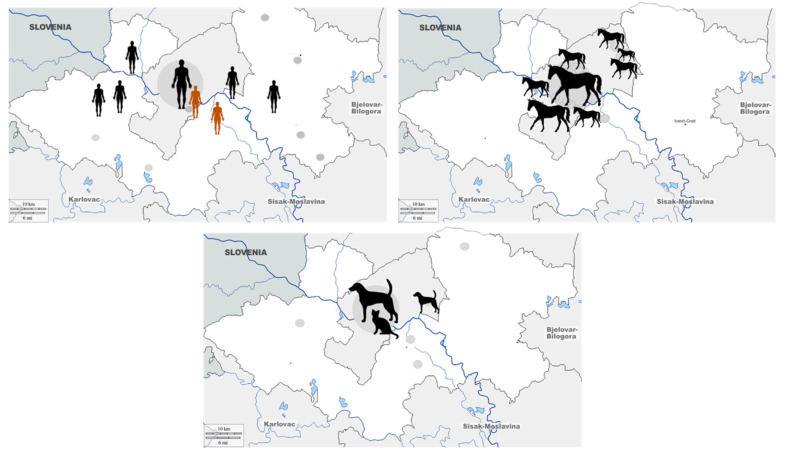
Geographic distribution of Tahyna virus seropositive humans, horses, and pet animals. (symbol size represents the number of cases).

The geographic distribution of seropositive humans and animals is presented in Figure 3. All seropositive humans were residents of urban areas. Among seropositive horses, 9 were from Zagreb city, and 20 were from suburban areas of Zagreb surroundings. In a group of pet animals, only one dog was from a suburban area.

**Figure 4:**
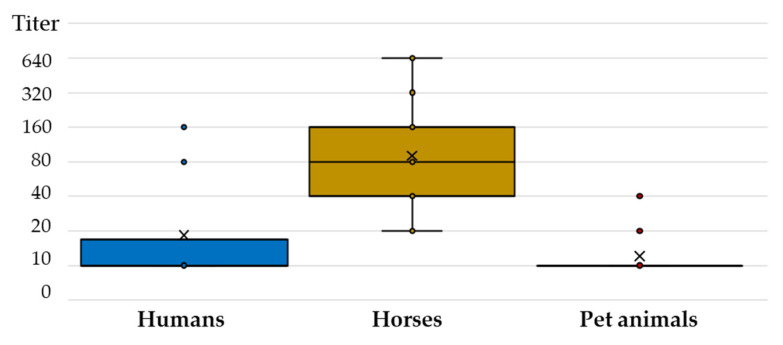
Tahyna virus-neutralizing antibody titers in humans and animals.

Comparing the TAHV NT antibody titers, a substantially higher titer was observed in horses (median 80, IQR = 40–160) compared to humans (median 10, IQR = 10–40) and pet animals (median 10, IQR = 10–10) (Figure 4).

A total of 4456 *Ae. vexans* mosquitoes were collected and identified in the Zagreb area, of which 4053/90.9% mosquitoes were sampled by traps with CO_2_, while 403/9.1% were sampled by the aspirator. Mosquito specimens were sorted in 159 pools and tested for the presence of TAHV (Table 5). All tested pools were negative for TAHV.

## 4. Discussion

Although TAHV is widely distributed in Europe, the number of clinical cases as well as the seroprevalence rates are probably underreported due to the lack of commercially available testing.

The frequency of TAHV NT antibody detection among patients with neuroinvasive disease from Zagreb and its surroundings was similar (9.4%) to a previous Croatian study (10.1%) conducted in patients from both continental and coastal regions. Only one published study, which was from the Sverdlovsk region (Russia) in 1994, analyzed the prevalence of TAHV in patients with encephalitis and found that up to 60% of patients had TAHV antibodies [32]. Therefore, the true prevalence and clinical significance of this neglected virus remain to be determined.

The TAHV seroprevalence rate in asymptomatic Croatian individuals was 3.7%, which was lower than the reported seroprevalence of 7.9% in the eastern region in the 1970s [23]. This difference should be explained at least in part by the studied population. This study included only inhabitants of a restricted urban area in northwest Croatia, while in the 1970s, individuals from a broad geographic area (northeastern continental and middle and south coastal area) were tested. However, the reported seroprevalence in this study was similar to the seroprevalence reported in some urban areas in Europe and Asia. In 2002, residents of an area in the Czech Republic affected by the flood were tested for the presence of antibodies to TAHV. While the highest seroprevalence rates of up to 28% were detected in rural and suburban populations of the forested floodplain along the Labe and Vltava Rivers, low seropositivity of 5% was found in the urban Prague area [33]. Two more recent studies showed lower TAHV seropositivity rates. A seroprevalence of 2% was recorded in Nasiriyah, the capital of the Dhi Qar Governorate in Southern Iraq, in 2012–2013 [34] In addition, TAHV NT antibodies were detected in 0.3% of blood donors from the Alpine Central European region of the Tyrol (North and East Tyrol, Austria, and South Tyrol, northern Italy) [18].

Outside Europe, very high TAHV NT seroprevalence rates ranging from 25% to 52% were recorded in the rural adult population of Cameroon in 2002–2003 [35]. In addition, epidemiological investigations showed high TAHV endemicity in both urban and rural areas in the Lao PDR. Several studies carried out in the communities of the Nakai plateau revealed a TAHV seropositivity of 30.45% in 2007 and 29.06% in 2010 using ELISA [36]. In patients presenting with fever with or without rash, the prevalence of TAHV antibodies was 13% using indirect immunofluorescence in an urban Kashi region, Xinjiang Province, China (2007) [37]. In the Lao PDR, TAHV antibodies were found in 37.7% of young patients under the age of 18 in 2015 [36].

The older age groups had a higher probability of infection and a higher prevalence of antibodies, and such a trend is usually seen in people and animals living in long-term enzootic regions [33]. Interestingly, in a Chinese study conducted at three locations in the Qinghai-Tibet Plateau, all seropositive individuals were under the 30-age group, with seroprevalence ranging from 1.1% to 4.3%. The authors supposed that higher seroprevalence in young age groups may suggest that TAHV was recently imported into the Qinghai-Tibet Plateau [21]. In the present study, no significant difference in seropositivity was found among age groups (2.5–7.7%).

Several studies have shown that TAHV circulates in wildlife in Central and Eastern Europe although seroconversion rates may vary by location and year. Several studies from the Czech Republic showed a declining trend in the TAHV seroprevalence among wild boars. The hemagglutination-inhibiting antibodies were found in 41.7% and 46.7% of wild boars in 1990 and 1993–1997, respectively [13,14]. A lower seropositivity rate of 19.4% was detected in the wild boars sampled at 24 hunting grounds of the Břeclav District (South Moravia) from 2000 to 2002. All these regions are characterized by the presence of wetland/fishpond ecosystems or floodplain forests as well as large mosquito populations [38]. In addition, TAHV NT antibodies were found in 28.9% of examined wild boars in Záhorská Lowland, western Slovakia, in the 1970s [39]. During 2016 and 2017, serum samples from wild boar (*Sus scrofa*), roe deer (*Capreolus capreolus*), and red deer (*Cervus elaphus*) were collected from Austria, Hungary, and Romania. In the wild boar population, TAHV NT seroprevalence rates were reported to be 25.9–27.5% in Austria, 0–55.6% in Romania, and 0–50.0% in Hungary. In addition, the seroprevalence in Austria was highest in wild boar (26.7%) compared to red deer (9.8%), while no roe deer were found seropositive [8].

TAHV seroprevalence in horses from urban areas of Zagreb and its surroundings tested in this study was 29.6%. This high seropositivity rate was similar to those detected in some endemic areas in the Czech Republic. In South Moravia (Břeclav District), a seroprevalence of 34.4% was reported in 1980. In addition, a very high seroprevalence rate of 55% was observed in pigs, while it was low in cattle (5.6%) [9]. In 2007, serum samples were collected from livestock (cows, sheep, and swine) at the abattoirs in Geermu City, Xining City, and Minhe County, China. In addition to IgG seropositivity of 6.7% in cows, 10.0% in sheep, and 3.3% in swine, IgM antibodies were detected in 3.3% of cows, 7.8% of sheep, and 5.0% of swine [21].

In this study, the TAHV seroprevalence rate in dogs and cats was 11.7%, with no difference according to sex and age. To our knowledge, there are no published data on the prevalence of TAHV in pet animals. In addition, there is no research on the clinical impact of TAHV infection in horses and pet animals. The results of this study clearly show that TAHV is circulating in selected animal species. There was additional reasoning for serosurvey of pet animals and horses. Compared to wildlife, serum samples are easier to collect. Moreover, unlike wildlife and other farm animals, these species are living in urban areas. Dogs and cats may live in close proximity to their owners and therefore have shared exposure to household and recreational risk factors. Finally, dogs and cats are scavengers, and pathogens bioaccumulate in them. The listed attributes make animal species selected for this study potential sentinel animals to monitor TAHV activity in cities. In analyzing the NT titers in humans and animals, substantially higher titers were observed in horses compared to humans and pet animals. Although there are no data regarding the longevity of TAHV antibody response in horses, it is still to be determined if higher titer makes them more sensitive tools for TAHV surveillance.

Many studies reported the detection of TAHV in mosquitoes. In 2006, TAHV was isolated from *Culex* spp. mosquitoes collected in Xinjiang, China [37]. Subsequently, in 2007–2008), TAHV was isolated from *Ae. dorsalis* and *Cx. modestus* pools in Inner Mongolia [20]. In 2013, the virus was isolated from *An. hyrcanus* mosquitoes collected on the fishponds in South Moravia (Czech Republic), a finding that represents the first isolation of TAHV from *An. hyrcanus* in Europe [7]. In a more recent study (2019), mosquitoes collected at floodplain habitats along three major rivers in eastern Austria, i.e., the Danube River, the Morava River, and the Leitha River, were tested. TAHV RNA was detected in two pools of *Ae. vexans* collected on the Leitha River. Phylogenetic analysis showed that the sequences obtained were remarkably similar to earlier TAHV isolates from the area, dating back to the initial TAHV isolate in 1958 [5]. In a very recently conducted study (2021), TAHV was detected in *Ae. caspius* and *Cx. pipiens* pools collected in the Emilia Romagna Region, Northern Italy. In addition, one isolated strain was obtained from one of the *Ae. caspius* pool collected in the municipality of Comacchio. Furthermore, TAHV was detected in 10 mosquito pools sampled in 2009, 2010, and 2020, confirming the continuous presence of the virus in this region [22]. In the present study, all tested *Ae. vexans* pools collected in the Zagreb area were negative for TAHV RNA.

When comparing the seropositivity rates between countries, it is important to keep in mind that the different serological methods (ELISA, IFA, and VNT) used to detect TAHV antibodies may have an impact on the seroprevalence results. In addition, there are some limitations of this study that should be noted. Since a small number of animals were included in the study, the seroprevalence results should be interpreted with caution. In addition, data on the sex and age of the horses were missing.

## 5. Conclusions

The presented results indicate the circulation of TAHV in northwestern Croatia. Further studies on large samples of humans, animals, and mosquitoes are needed to determine the prevalence of this neglected viral zoonosis.

## Figures and Tables

**Table 1:** Tahyna virus seroprevalence in Zagreb and the surrounding area.

Study Population		N Tested	N (%) VNT-Positive	95% CI
Humans	Patients with neuroinvasive disease	32	3 (9.4)	1.9–25.0
	Asymptomatic individuals	218	8 (3.7)	1.6–7.1
Animals	Horses	98	29 (29.6)	20.8–39.6
	Pet animals	94	11 (11.7)	5.9–19.9

VNT, virus-neutralization test; CI, confidence interval.

**Table 2:** Tahyna virus seroprevalence in asymptomatic individuals according to demographic characteristics.

Characteristic		N (%) Tested	N (%) Positive	95% CI	*p*
Sex	Male	108 (49.5)	4 (3.7)	1.0–9.2	0.978
	Female	110 (50.5)	4 (3.6)	1.0–9.1	
Age	<30 years	33 (15.1)	1 (3.0)	0.1–15.7	0.677
	30–49 years	79 (36.3)	2 (2.5)	0.3–8.8	
	50–69 years	80 (36.7)	3 (3.8)	0.8–10.6	
	70+ years	26 (11.9)	2 (7.7)	0.9–25.1	
Area of residence	Urban	192 (88.1)	8 (4.2)	1.8–8.0	0.288
	Suburban	26 (11.9)	0 (0)	0–13.2 *	

* One-sided 97.5% confidence interval; CI, confidence interval.

**Table 3:** Risk analysis for Tahyna virus seropositivity.

Characteristic	OR	95% CI OR	*p*	RR	95% CI RR	*p*
Male (Ref.) vs. female sex	1.019	0.248–4.013	0.978	1.018	0.261–3.969	0.978
Age						
<30 years	Ref.			Ref.		
30–49 years	0.831	0.072–9.494	0.881	0.835	0.078–8.900	0.881
50–69 years	1.246	0.124–12.441	0.851	1.237	0.133–11.469	0.851
70+ years	2.666	0.228–45.547	0.782	2.538	0.243–26.480	0.436
Suburban (Ref.) vs. urban area of residence	2.441	0.136–4.183	0.543	2.378	0.141–40.045	0.547

OR, odds ratio; CI, confidence interval; RR, relative risk.

**Table 4:** Seroprevalence of Tahyna virus in pet animals according to sex and age.

Characteristic		Dogs				Cats		
	N (%) Tested	N (%) Positive	95% CI	*p*	N (%) Tested	N (%) Positive	95% CI	*p*
Sex				0.713				0.533
Male	37 (52.8)	5 (13.5)	4.5–28.7		15 (62.5)	1 (6.7)	0.2–31.9	
Female	33 (47.2)	3 (9.1)	1.9–24.3		9 (37.5)	2 (22.2)	2.8–60.0	
Age				0.675				0.115
<5 years	19 (27.5)	3 (15.8)	3.4–39.6		9 (40.9)	2 (22.2)	2.8–60.0	
>5 years	50 (72.5)	5 (10.0)	3.3–21.8		13 (59.1)	1 (7.7)	0.2–36.0	

**Table 5:** Number of collected *Aedes vexans* mosquitoes/pools tested for Tahyna virus.

Sampling Method	N Collected Specimens/N Pools Tested for the Presence of TAHV			
Year	2020	2021	2022	Total
CDC Mini Light trap	1414/44	2215/66	424/18	4053/128
Aspirator	266/18	119/12	18/1	403/31
Total	1680/62	2334/78	442/19	4456/159

## Data Availability

Not applicable.
